# Role of cytopathology in diagnosing phaeohyphomycosis masquerading as nerve abscess in a lepromatous leprosy patient: A case report

**DOI:** 10.1016/j.ijscr.2023.108741

**Published:** 2023-08-29

**Authors:** Shakti Kumar Yadav, B.K. Chandana, Hemlata Panwar, Jai Kumar Chaurasia, E. Jayashankar, Dinesh Asati

**Affiliations:** a Department of Pathology and Laboratory Medicine, All India Institute of Medical Sciences, Bhopal, India; b Department of Dermatology, All India Institute of Medical Sciences, Bhopal, India

**Keywords:** Phaeohyphomycosis, Lepromatous leprosy, Fine needle aspiration cytology, Immunocompromised, Melanized hyphae

## Abstract

**Introduction and importance:**

Phaeohyphomycosis is a rare fungal infection primarily affecting immunocompromised individuals. Its clinical manifestations are diverse, and diagnosis can be challenging, particularly when lesions mimic other conditions.

**Case presentation:**

A 66-year-old male, with a history of irregular leprosy treatment and prolonged steroid use, presented with symptoms suggestive of a nerve abscess. On examination, cystic swellings were observed on the left thumb and leg. Histopathological examination and fine needle aspiration cytology (FNAC) revealed melanized hyphae, leading to a final diagnosis of phaeohyphomycosis. The patient was treated with oral itraconazole, leading to regression in lesion size.

**Clinical discussion:**

Leprosy patients on long-term steroids are especially susceptible. The pathogenicity of these fungi in immunocompetent people is believed to be due to melanin in their cell walls, which defends against host defenses. Diagnosis involves histopathological examinations, staining, and fungal culture. Treatment involves surgical excision and antifungal drugs. If untreated, it can lead to severe complications including fatal brain infections.

**Conclusion:**

This case highlights the unusual presentation of phaeohyphomycosis mimicking a nerve abscess in a leprosy patient. It underscores the importance of a high degree of clinical suspicion in diagnosing such rare infections, particularly in immunocompromised individuals. It also emphasizes the value of FNAC in reaching a definitive diagnosis. Prompt diagnosis and appropriate treatment are essential to prevent potentially serious outcomes.

## Highlights

• A high suspicion is required, as this can mimic conditions, such as nerve abscesses, particularly in patients with leprosy.
• Prolonged use of steroids and immunosuppressive conditions, like leprosy, can predispose patients to phaeohyphomycosis.
• Further research into the pathogenic mechanisms of these fungi, especially in immunocompetent individuals is needed.
• FNAC with special stains in aspirated material can be pivotal for diagnosis.
• Treatment involves surgical excision for cutaneous and subcutaneous lesions and medical management using antifungal drugs.

## 1 Introduction

Phaeohyphomycosis is a rare subcutaneous and cutaneous fungal infection caused by dematiaceous fungi. Rarely verrucous plaques and pustules can occur in phaeohyphomycosis. Pleomorphic and widespread lesions in a single patient are rare and are usually seen in immunocompromised cases [1].

Subcutaneous Phaeohyphomycosis, a rare form of deep mycosis associated with cystic swellings involving the extremities, is caused by melanized saprophytic fungi present ubiquitously in the environment [2]. Rarely it can co-exist with leprosy. The most common clinical presentations are chromoblastomycosis and phaeohyphomycosis. Chromoblastomycosis is characterized by subcutaneous nodules, plaques, and tumors, while phaeohyphomycosis presents subcutaneous abscesses, cellulitis, and mycetoma [3].

The disease is reported worldwide, but the incidence and causative agents vary by region. In tropical and subtropical regions, the most common causative agents are *Cladosporium* species, *Fonsecaea pedrosoi*, and *Phialophora verrucosa*. In temperate regions, the most common causative agents are *Alternaria*, *Bipolaris*, and *Curvularia* species. In these regions, Phaeohyphomycosis mainly affects healthy individuals and is often associated with traumatic implantation of the fungus [4].

In India, the disease is primarily reported in immunocompromised individuals, particularly those with diabetes and HIV/AIDS. A study from India reported that the most common causative agents of Phaeohyphomycosis in India are *Cladosporium* species, followed by *Fonsecaea pedrosoi* and *Phialophora verrucosa*. The majority of cases were reported from the southern and eastern regions of the country, with a higher incidence in males [5]. Another study conducted in India found that the majority of Phaeohyphomycosis cases were in patients with diabetes (60.5 %) and HIV/AIDS (35.9 %). The most common clinical presentations were subcutaneous abscesses and nodules (57.1 %), followed by mycetoma (22.9 %) [6].

We report a unique presentation of the disease mimicking a nerve abscess on leg in a case of lepromatous leprosy, diagnosed by fine needle aspiration cytology (FNAC). In Indian context, this case report highlights the importance of appropriate treatment and follow up of leprosy patients especially when associated with addition morbidities. This case is reported in line with the SCARE criteria [7].

## 2 Case report

A 66-year-old male, farmer, chronic smoker, presented with multiple erythematous evanescent tender nodules over body associated with fever and joint pain for 25 years. He also had multiple skin colored shiny infiltrated umbilicated nodules over earlobe, trunk and extremities with glove and stocking anesthesia (up to wrist and ankle), bilateral partial ulnar claw hand and trophic ulcers on foot.

He was a defaulter with irregular treatment with anti-leprosy drugs for past 20 years and had been self-medicating with varying doses of oral steroids for the reactional episodes.

The hematological, biochemical and serological profile of this patient was within normal range. However, the patient exhibited hyperglycemia with a fasting glucose level of 120.36 mg/dL.

**Fig. 1:**
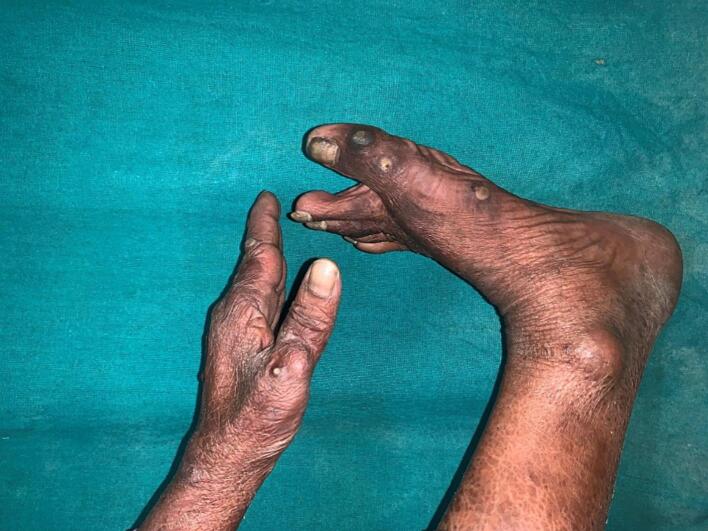
Two ill-defined skin-colored cystic swellings involving extremities.

**Fig. 2:**
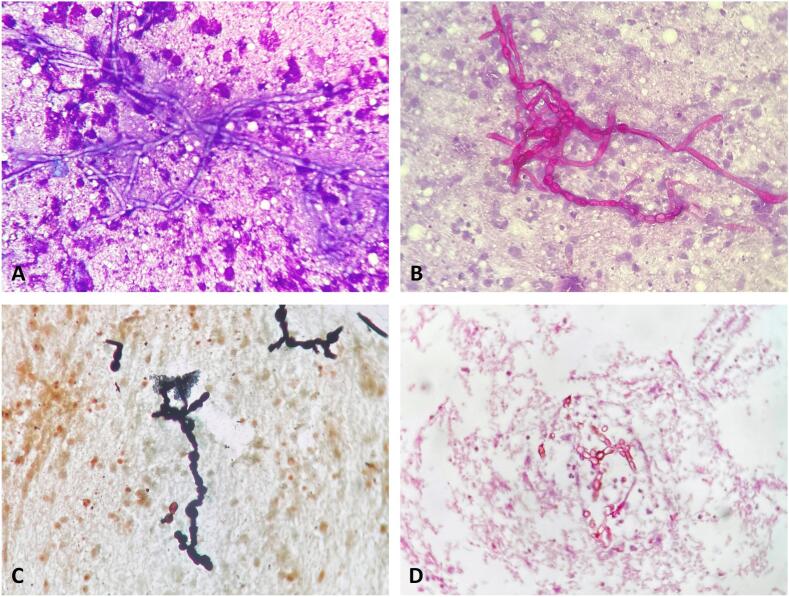
Smear showing septate toruloid hyphae (A-MGG stain, B-PAS Stain and C-Masson’s Fontana stain, 400x). Cell block preparation showing the fungal hyphae (PAS, 400x).

**Fig. 3:**
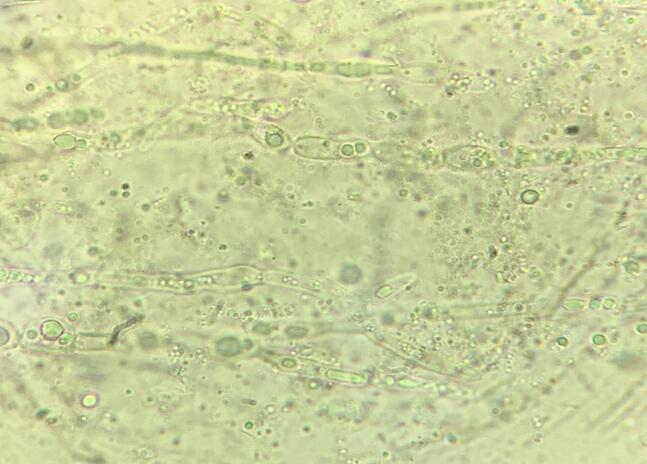
10 % KOH mount showing hyaline septate toruloid hyphae with constriction at site of septation with multiple inclusions and scant melanisation in aspirate from left ankle swelling.

On examination two well defined mobile, fluctuant cystic swellings were present on left thumb and left leg above the medial malleolus measuring 1 × 1 cm and 9 × 7 cm respectively for 6 months (Fig. 1). They were mildly tender on palpation, followed the course of nerves. The routine blood investigations were within normal range except leukocytosis and finding of chronic obstructive lung disease on chest X-ray. Slit skin smear from ear nodules was positive for Wade fite stain for lepra bacilli (+6). Biopsy from the skin papules was consistent with lepromatous leprosy. With differentials of nerve abscess and cutaneous cysticercosis, FNAC of the nodules was done. FNAC smears showed scattered population of few viable and many degenerated mixed inflammatory cells, necrotic material and many fungal hyphae. These were broad bulbous hyaline to melanized having constrictions at septal region (Fig. 2A). These fungal hyphae stained positively with PAS and Masson Fontana (Fig. 2B, C). Fungal hyphae were also highlighted on 10 % potassium hydroxide mount (KOH) preparation (Fig. 3) which, however the fungal culture came out be negative in this case.

**Fig. 4:**
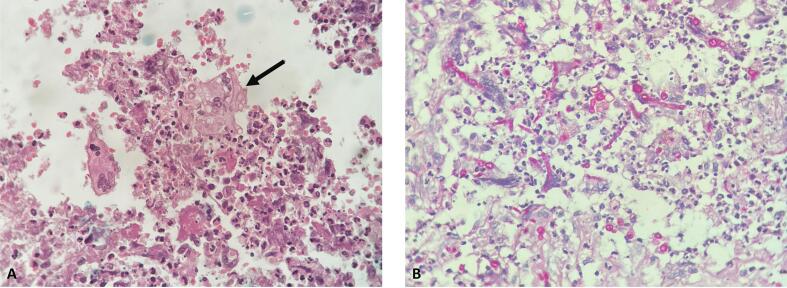
Section from the excised cyst showing septate fungal hyphae (black arrow) along with necrotic debris, foreign body giant cells, foamy histiocytes, neutrophils and eosinophils (A, H&E, 400×). PAS stain highlighting the fungal hyphae (B, PAS, 400×).

Surgical excision of the cysts was done under antifungal cover which showed fibrous walled off cyst with necrotic center containing multiple broad septate toruloid hyphae with multiple foreign body giant cells and mixed inflammatory infiltrate, fungal hyphae stained with PAS stain (Fig. 4). With a final diagnosis of phaeohyphomycosis, patient was started on oral itraconazole 200 mg leading to regression in lesion size. Excision site healed well however patient was lost to follow over next few months.

## 3 Discussion

Phaeohyphomycosis is described as infection by dematiaceous fungi of *Exophiala, Alternaria, Cladophialophora, Lomentospora* and *Curvularia* genera [8]. Cutaneous involvement can present as papules, nodules, non-healing ulcers, abscess, cysts and sinues [1]. Patients with immunosuppression (primary or secondary), residence in tropical and temperate climates and agricultural work are predisposed to infection. Certain genera such as *Cladophialophora* and *Rhinocladiella,* and *Exophiala* and *Verruconis* show neurotropism in immunocompetent and immunosuppressed patients respectively [8].

Leprosy patients of any spectrum with prolonged use of steroids predisposes them to this infection with duration ranging from six months to four years and the highest total cumulative dose being 12.6 g [9].

Patients may often give a history of prior trauma which was not present in our case. Co-existence of lepromatous leprosy and subcutaneous phaeohyphomycosis is not an unusual occurrence due to immunosuppressed (iatrogenic or genetic) state associated in both diseases.

CARD9 mutations have been implicated in predisposition to these infections 4–6. CARD 9 mutation analysis could not be done due to unavailability of the assay in our setup. Other causes of immunosuppression were ruled out through a normal complete blood count and negative retroviral serology [10].

The pathogenic mechanisms by which these fungi cause disease, particularly in immunocompetent individuals, remain largely unknown. One of the likely candidate virulence factors is the presence of melanin in the cell wall, which is common to all dematiaceous fungi. Melanin is thought to confer a protective advantage by scavenging free radicals and hypochlorite that are produced by phagocytic cells in their oxidative burst and that would normally kill most organisms. In addition, melanin may bind to hydrolytic enzymes, thereby preventing their action on the plasma membrane. These multiple functions may help explain the pathogenic potential of some dematiaceous fungi, even in immunocompetent hosts [11].

Cystic lesions commonly encountered in leprosy are nerve abscess, or bone cyst and phaeohyphomycotic cysts rarely [1,9,12]. FNAC with special staining helps in confirming diagnosis. In phaeohyphomycosis, aspirate reveals melanized toruloid septate fungal hyphae as noted in *Exophiala*. They require excision and antifungal therapy [1,13].

Histopathological examination of lesions shows abscess cavity, with granulomatous inflammatory cells, epithelioid cells admixed with eosinophils, lymphocytes, and multinucleated giant cells with fungal hyphae. These hyphae were better visualized on periodic acid–Schiff and silver methenamine staining. The Masson-Fontana stain (MF), which is specific for melanin, stains the fungal profiles brownish black [11].

Aspirated material from the cystic cavity can be sent to fungal culture, with Sabouraud dextrose agar, will reveal septate branching hyphae and show yeast-like dark-pigmented fungal colonies after 5–7 days of incubation both at 28 °C and 37 °C [11]. However the fungal culture was negative in our case.

The treatment modalities depend on the site of infection. Surgical excision for cutaneous and subcutaneous lesions presenting as like abscess and cyst. This is supplemented with medical management using antifungal drugs like Itraconazole, Voriconazole and Amphotericin B. Prolonged therapy may be required for more disseminated disease. If untreated this can lead to deep inoculation into the subcutaneous soft tissues, can cause rhinosinusitis, and even cerebral phaeohyphomycosis, which can be fatal [11,14].

This case underlines the fact that leprosy patient with co-morbities and immunocompromised conditions should be closely followed up to prevent such complications. Prevalence for such fungal infections in patient undergoing treatment for leprosy may be further investigated to determine the risk factors and etiopathogeneis of phaeohyphomycosis in leprosy.

## 4 Conclusion

Phaeohyphomycosis, a rare fungal infection, can present with varied clinical manifestations and is prevalent particularly among immunocompromised individuals. This case study sheds light on a unique presentation of phaeohyphomycosis in a leprosy patient, furthering our understanding of its potential clinical complexity.

## Consent

Written informed consent was obtained from the patient for publication of this case report and any accompanying images.

## Ethical approval

Ethical approval not required, as case reports are exempted from ethical approval in authors' institute.

## Funding

No funding has been received.

## Author contribution

Drafting the manuscript and Literature research: SKY and CBK.

Diagnostic workup: SKY, EJ, HP, JKC.

Treatment planning: DA.

Supervision and critical revision: SKY.

## Guarantor

Shakti Kumar Yadav.

## Research registration number

1. Name of the registry: N/A
2. Unique identifying number or registration ID: N/A
3. Hyperlink to your specific registration (must be publicly accessible and will be checked): N/A

## Conflict of interest statement

None declared.
